# Efficiency of turmeric, triphala and aloe vera in the treatment of oral submucous fibrosis

**DOI:** 10.6026/973206300210630

**Published:** 2025-04-30

**Authors:** Saikiran Bahadur, Santosh T., Omar Basheer Altaher Mohammed, Salma F., Deepak T.S, Bhavana Agrawal, Deepa E., Sneha Saha

**Affiliations:** 1 Private Practitioner, Departments of Prosthodontics, Springfield Dental, Springfield, Massachussets, United State of America; 2 Department of Oral Pathology & Microbiology, Government Dental College, Dibrugarh, Assam, India; 3 Department of Oral and Maxillofacial Surgery, Sebha Dental Faculty, Sebha University, Lybia, Country in North Africa; 4 Department of Oral and Maxillofacial Pathology, NIMS University, Jaipur, Rajasthan, India; 5 Department of Oral Medicine and Radiology, Subbaiah Institute of Dental Sciences, Shivamogga, Karnataka, India; 6 Department of Oral Medicine and Radiology, Pacific Dental College and Hospital, Udaipur, Rajasthan, India; 7 Department of General Pathology, SRM Dental College, Ramapuram, Bharathi Salai, Chennai, Tamil Nadu, India; 8 Intern, Kalinga Institute of Dental Sciences, Bhubaneswar, Odisha, India

**Keywords:** Aloe vera, oral submucous fibrosis, triphala, treatment

## Abstract

Oral Submucous Fibrosis (OSMF) is a condition marked by progressive fibrosis of the oral mucosa, which makes difficulty in opening mouth and causes other painful symptoms. Conventional treatment options for Oral Submucous Fibrosis are limited and their effectiveness varies. Hence, herbal formulations have gained attention as alternative and complementary treatment options for Oral Submucous Fibrosis. Therefore, it is of interest to assess the efficiency of herbal medicine in the treatment of Oral Submucous Fibrosis 40 patients diagnosed with Oral Submucous Fibrosis were equally alienated into 4 groups of herbal medicine for the management as; Group I-turmeric, Group II-triphala, Group III- aloe vera and group IV- control placebo group. Each patent were asked to apply particular medicine two times daily for 3 months. The efficacy was checked for reduction in burning sensation and improvement in mouth opening. There was improvement in mouth opening, decrease in burning sensation and pain after the treatment. Triphala was found efficient in improvement of Oral Submucous Fibrosis symptoms followed by turmeric and aloe Vera.

## Background:

A potentially cancerous condition known as oral submucous fibrosis (OSMF), which has increasing fibrosis of the oral mucosa with limited mouth opening, mucosal stiffness and difficulty in eating and speaking [1]. A juxta-epithelial inflammatory response and increasing fibrosis of the submucousal tissues (the lamina propria and deeper connective tissues) are the hallmarks of this chronic, complicated precancerous condition of the mouth [2]. In India, oral submucous fibrosis (OSF) is more common. According to reports, the prevalence of Oral Submucous Fibrosis varies by gender in India, ranging from 0.2-2.3% in men to 1.2-4.5% in women [3]. The total prevalence rate is estimated to be between 0.2-0.5percent. According to estimates, tobacco-related ailments claim the lives of nearly 1 million Indians each year [4, 5]. Although the OSMF's etiopatho-genesis is still unclear; it is known to have a complex aetiology. Chronic chewing of areca nuts, the main ingredient in betel quid, is known to produce this condition. The areca nut's constituent causes increased reactive oxygen species, which damages cellular structures. Malnutrition and iron and vitamin deficiencies accompany this [6]. In addition to other factors like immunological dysregulation, genetic susceptibility and nutritional inadequacies, Oral Submucous Fibrosis is frequently linked to tobacco use and the habit of chewing areca nuts [7]. Frequent chewing and contact with these materials can cause long-term irritation and inflammation of the oral tissues, which can ultimately result in the development of fibrous bands and a narrowed mouth opening. Eating, speaking and other oral functions can all be severely hampered by this illness [8]. A delayed diagnosis may result from the ease of ignoring the early signs of Oral Submucous Fibrosis which include nonspecific vesicular stomatitis and a burning feeling in the oral cavity.

Vertical bands that run through the mucosa form as the disease worsens and the distinctive fibrosis of the cheeks, lips, tongue, or palate becomes noticeable. The oral mucosa becomes rigid and less elastic due to the fibrotic alterations, causing trismus, or trouble opening the mouth, which can significantly impair a person's capacity to speak and eat. Significant limitations in opening the mouth and reduced mobility of the tongue and/or palate can result from the severity of the fibrosis progressing to the point where the affected parts appear white and rigid. An individual's quality of life may be significantly impacted by this [8]. The illness progresses over time and the clinical manifestations vary according to the disease's stage. Most patients have a spicy food intolerance and lip, tongue and palate rigidity, which limit their ability to open mouth and to move their tongues [2]. Muscle tonicity is diminished in the masticatory and facial expressive muscles [9]. The management and prevention of problems connected to Oral Submucous Fibrosis can be greatly improved by prompt intervention, lifestyle changes and quitting tobacco products [8]. The most widely used treatment for Oral Submucous Fibrosis to date is steroids, but the truth is that drugs have some drawbacks [9]. The recommended medicinal and surgical treatment techniques for symptom relief have not yet proven effective. Other methods are also suggested such as surgical excision, laser removal, *etc.* However, each treatment has its own limitations [6]. The success of the few conventional therapy options for Oral Submucous Fibrosis varies. Hence herbal formulations have gained attention as alternative and complementary treatment options for Oral Submucous Fibrosis In order to treat Oral Submucous Fibrosis and encourage wound healing, numerous natural plant extracts, herbal extracts, synthetic medications, *etc.*, have been presented and tested [10]. The rationale behind the use of herbal formulations in Oral Submucous Fibrosis lies in the presence of bioactive constituents in plants that exhibit pharmacological activities, such as anti inflammatory, antioxidant, anti-fibrotic and immuno-modulatory effects [7]. The use of herbal formulations in Oral Submucous Fibrosis treatment has gained attention due to their potential therapeutic effects, minimal side effects and perceived natural origin. Herbal formulations for Oral Submucous Fibrosis typically consist of various plant derived ingredients that possess bioactive compounds with potential therapeutic properties. Numerous herbal ingredients have been investigated for their potential efficacy in Oral Submucous Fibrosis treatment, including Aloe vera, curcumin, licorice, Trikatu, honey, Triphala, turmeric, Sesame oil, Green tea [6, 11].

Vitamins A, C, E, B1, B2, B3, B6, choline, folic acid, beta-carotene and B12 are all found in aloe vera, a plant also known as "wound-healing hormone" [12]. Anti-inflammatory, wound-healing, immuno-modulatory, antioxidant and anti-tumor activities are all present in Aloe Vera gel. Aloe vera's many qualities raise the prospect of using it to treat oral submucous fibrosis. In Oral Submucous Fibrosis it has been applied topically or as a mouthwash to lessen inflammation and alleviate symptoms [13]. Triphala is a combination of three fruits amla, haritaki and bibhitaki used in traditional Ayurveda medicine. It possesses antioxidant, anti inflammatory and immuno-modulatory effects [7]. Curcumin, demethoxycurcum in and bisdemethoxycurcum in are the three curcuminoids found in turmeric, one of the popular natural products. Turmeric has anti-mutagenic properties and improves blood circulation [2]. Turmeric contains a bioactive substance called curcumin, which has anti-inflammatory, anti-fibrotic and antioxidant qualities. In Oral Submucous Fibrosis it has been investigated for its capacity to prevent fibrosis, lessen inflammation and enhance oral mucosal health [7, 11]. Herbal medications' anti-inflammatory, antioxidant, anti-stress and analgesic qualities are the suggested mode of action in Oral Submucous Fibrosis [14]. There are very few studies done on comparison of various herbal medicines in management of Oral Submucous Fibrosis. Therefore, it is of interest to evaluate the efficacy of herbal medicine (turmeric, triphala, aloe vera) in the treatment of Oral Submucous Fibrosis.

## Materials and Methods:

After receiving ethics committee permission and informed consent from each participant, this prospective interventional study was conducted at the oral medicine department. The study comprised participants from the outpatient department who had chronic Oral Submucous Fibrosis which is characterised by pain in the oral cavity at rest, mucosal blanching and difficulty in opening the mouth. Participants with a history of allergies or severe systemic diseases were not allowed to participate in the trial. The study was conducted by a qualified researcher. The current investigation included 40 patients who had been clinically and histo-pathologically diagnosed with Oral Submucous Fibrosis. The subjects are randomly divided equally into 4 groups with 10 samples in each group as; Group I-turmeric, Group II-triphala, Group III- aloe vera and group IV- control placebo group. We bought turmeric root stem and triphala powder from the local market. Turmeric was grind to fine powder. To make a paste, one gram of triphala or turmeric powder was mixed with glycerine. The aloe vera gel was obtained from fresh aloe vera leaf. Each patent were asked to apply particular medicine two times daily for 10 minutes before eating or drinking for 3 months. The efficacy was checked for improvement in mouth opening, decrease in burning sensation.

In order to evaluate the mouth opening, the distance between the incisal margins of the upper central incisors and the lower central incisor at maximum jaw opening was measured in millimetres (mm) before and after treatment. A visual analogue scale (VAS) was used to quantify the burning sensation score before and after treatment. A score of 0-1 indicated absence, a score of 1-6 indicated reduction and a score of 7-10 indicated severity. For three months, all patients were recalled at one-month intervals. Following the procedure, no biopsy was performed. Patients receiving the aforementioned treatment did not experience any side effect that was clinically noteworthy. Every patient was encouraged to change their lifestyle and given advice on how to stop alcohol and tobacco products. The obtained data statistically analysed using SPSS 22.0 (SPSS Inc., Chicago, IL, USA) using the ANOVA test. P < 0.05 was considered statistically significant.

## Results:

The average age of the 32 males and 8 females was 30.4 ± 3.5 years. In herbal groups, there was a reduction in the burning feeling and an improvement in mouth opening. The control group did not see any changes in burning or mouth opening (Table 1, Table 2). Triphala was effective in improving the Oral Submucous Fibrosis symptoms followed by turmeric and aloe vera. The tested herbal medications can be suggested to treat cases of oral submucous fibrosis.

## Discussion:

Herbal preparations have drawn interest as possible Oral Submucous Fibrosis therapy alternatives. Any treatment modality must include stopping of chewing areca or betel quid. In the current investigation, we discovered that using herbal medications improved mouth opening and reduced burning feeling. Srivastava *et al.* investigated the clinical efficacy of herbal remedies (1 gmtulsi and 1 gm turmeric mixed in glycerine base) for the treatment of oral submucous fibrosis (OSMF). They came to the conclusion that turmeric and tulsi are a safe and effective way to treat Oral Submucous Fibrosis symptoms [11]. The usefulness of honey, tulsi (holy basil) and turmeric in treating Oral Submucous Fibrosis was compared by Mobeen *et al.* The mouth opening, tongue protrusion, burning sensation, mucous membrane blanching and decrease in palpable fibrous bands were all significantly altered, according to the author [8]. Tulsi and turmeric together can be regarded as a superior option for managing all grades of Oral Submucous Fibrosis according to Virani *et al.*'s evaluation of the two remedies in the treatment of oral submucous fibrosis [2]. Aich *et al.* demonstrated the safety and efficiency of a mouthwash containing honey, triphala and curcumin in reducing Oral Submucous Fibrosis symptoms [15]. The burning and mouth opening sensations were shown to have improved statistically significantly by Singh *et al.* For the treatment of Oral Submucous Fibrosis symptoms, a natural product combination consisting of triphala and turmeric is safe and efficient [16]. Bohra *et al.* compared the effectiveness of steroid therapy with Kali haldi in the treatment of oral submucous fibrosis (OSMF). According to the study's findings, combination therapy is quite effective for Oral Submucous Fibrosis [9].

According to Dalai *et al.* research, aloe vera and tulsi offer a safe and efficient way to treat Oral Submucous Fibrosis symptoms [12]. According to a study by Anuradh *et al.* aloe vera may be a safe, effective and alternative therapy option for oral submucous fibrosis [13]. According to Chen *et al.* comprehensive review, aloe vera is the most effective remedy for severe burning sensation complaints, while lycopene has little adverse effects. More high-quality RCTs are required to find better treatments for OSF, even though the latest review has made some progress. Drug therapy for OSF is still uncertain [17]. According to Patel *et al.* an ayurvedic therapy plan is safe and effective for managing Oral Submucous Fibrosis [18]. The main ingredient in curcuma longa, curcumin, is what gave turmeric its bulk of its medicinal properties. Due to its anti-inflammatory, analgesic and antioxidant qualities, turmeric have also been utilised extensively [15]. Singh *et al.* discovered that using Triphala and turmeric to treat Oral Submucous Fibrosis cases significantly reduced the burning and improves mouth opening [16]. According to Ritesh *et al.* mouthwashes that contain honey, triphala and curcumin are useful in reducing Oral Submucous Fibrosis symptoms [19]. In order to reduce the clinical signs and symptoms of Oral Submucous Fibrosis patients, Bhelonde *et al.* evaluated the effectiveness of topical application of a new gel combining curcumin, Aloe vera, honey and physiotherapy with 0.1% triamcinolone acetonide and physiotherapy. They came to the conclusion that Novel Gel reduces Oral Submucous Fibrosis symptoms just as well as topical steroid triamcinolone acetonide [20]. Singh *et al.* came to the conclusion that the combined effects of aloe vera paste and turmeric lead to a more effective and powerful treatment for oral submucosal fibrosis [21]. Rajbhoj *et al.* found that while both aloe vera gel and curcumin gel can effectively reduce Oral Submucous Fibrosis symptoms, aloe vera gel is more efficient in reducing burning sensations [22]. Following the use of aloe vera and curcumin medications, Rizvi *et al.* saw an improvement in symptoms associated with Oral Submucous Fibrosis [23]. Even though herbal formulations are usually regarded as safe, they can nonetheless have negative consequences, especially when taken in excess or improperly. The smaller sample size and the frequently sparse and inconsistent data base supporting the effectiveness of herbal formulations in treating Oral Submucous Fibrosis are the study's limitations.

## Conclusion:

Triphala was effective in improving the Oral Submucous Fibrosis (OSMF) symptoms followed by turmeric and aloe vera. The tested herbal medications can be suggested to treat cases of Oral Submucous Fibrosis (OSMF).

## Figures and Tables

**Table 1:** Improvement in mouth opening

**Group**	**Baseline Mean ±SD**	**After 1 month**	**After 3 months**	**p**
Group I Turmeric	23.24±2.56	25.34±	27.42±	0.001
Group II Triphala	23.01±2.45	24.34±	28.12±	0.001
Group III aloe vera	23.23±2.42	24.34±	27.23±	0.001
Group IV- plecebo	23.14±2.32	22.21±	22.34±	0.86

**Table 2:** Improvement in burning sensation (VAS)

**Group**	**Baseline Mean ±SD**	**After 1 month**	**After 3 months**	**p**
Group I Turmeric	6.12±1.23	4.46±0.43	0.34±0.32	0.001
Group II Triphala	6.12±1.23	4.64±0.34	0.23±0.21	0.001
Group III aloe vera	612±1.23	4.55±0.37	0.42±0.22	0.001
Group IV- plecebo	5.12±1.23	5.34±0.33	5.45±0.53	0.76
VAS: Visual analogue scale
